# 256 Stratifying Suspect Measles Cases: Development and Evaluation of an Infection Prevention Response Matrix

**DOI:** 10.1017/ash.2026.10732

**Published:** 2026-06-23

**Authors:** Florence Whitehill, Amanda Lyons, Bethelhem Abera, Colin Adler, Maria Burgos-Garay, Mariya Campbell, Jamari Moore, Ariel J. Santiago, Christine Ganim, Yimu Cahela, Susanna Lenz, Paige Gable, Magdalena Medrzycki, Maroya Walters, Amelia Keaton, Angela Coulliette-Salmond

**Affiliations:** 1 CDC; 2 Centers for Disease Control and Prevention; 3 CDC, U.S. Public Health Service

## Abstract

**Background:** Healthcare facility-level wastewater surveillance (WWS) is a relatively novel tool for pathogen surveillance. The most sensitive methods for detection of severe acute respiratory syndrome coronavirus 2 (SARS-CoV-2) in wastewater remain unknown. In this study at skilled nursing facilities (SNFs), two wastewater concentration methods were evaluated for SARS-CoV-2 detection by droplet digital polymerase chain reaction (ddPCR): Electronegative membrane filtration (enMF) and Nanotrap® Magnetic Virus Particles (NP). SARS-CoV-2 case surveillance data from the SNFs was incorporated into this evaluation. **Methods:** Wastewater samples were collected weekly from manholes at three SNFs over 25 weeks in 2022. Samples were split and concentrated by both enMF and NP, then virus concentration was determined by ddPCR. We utilized a weekly questionnaire and each facility’s National Healthcare Safety Network COVID-19 Report to identify the number of resident and healthcare personnel cases in each facility. Differences in the median SARS-CoV-2 concentration were compared across the enMF and NP methods using the Wilcoxon signed-rank test (?=0.05). Spearman’s correlation (?) was used to evaluate the relationship between the SARS-CoV-2 concentration in wastewater and SARS-CoV-2 case counts. **Results:** For each facility separately, as well as overall, median virus concentration in wastewater from enMF was higher than that from NP (SNF A: 2.84 vs. 1.49 genome copies [gc]/100 mL; SNF B: 8.50 vs. 6.08 gc/100 mL; SNF C: 5.3 vs. 3.86 gc/100 mL; overall: 5.3 vs. 3.86 gc/100 mL). The Wilcoxon signed-rank test confirmed that the difference in median virus concentrations was statistically significant for SNF B (p=0.001) and overall (p=0.001). Spearman’s correlation results showed overall higher correlations with enMF than with NP and a greater number of statistically significant results among subpopulations. For example: using enMF, the correlation among residents and healthcare personnel combined at SNF A was ?=0.86 (p=<0.0001), at SNF B was ?=0.50 (p=<0.01), and at SNF C was ?=0.59 (p=<0.01), whereas using NP, the correlation at SNF A was ?=0.54 (p=<0.01), SNF B was ?=0.46 (p=0.02), and SNF C was ?=0.23 (p=0.26). **Conclusions:** enMF yielded higher SARS-CoV-2 concentrations than NP and produced stronger correlations between wastewater signal and case counts, though both methods yielded acceptable results. enMF should be considered as an effective virus concentration method for healthcare facility-level WWS to strengthen and complement clinical methods of case detection.

